# Antimicrobial Chemicals Associate with Microbial Function and Antibiotic Resistance Indoors

**DOI:** 10.1128/mSystems.00200-18

**Published:** 2018-12-11

**Authors:** Ashkaan K. Fahimipour, Sarah Ben Mamaar, Alexander G. McFarland, Ryan A. Blaustein, Jing Chen, Adam J. Glawe, Jeff Kline, Jessica L. Green, Rolf U. Halden, Kevin Van Den Wymelenberg, Curtis Huttenhower, Erica M. Hartmann

**Affiliations:** a Biology and the Built Environment Center, University of Oregon, Eugene, Oregon, USA; b Department of Civil and Environmental Engineering, Northwestern University, Evanston, Illinois, USA; c Biodesign Center for Environmental Health Engineering, Biodesign Institute, Arizona State University, Tempe, Arizona, USA; d Energy Studies in Buildings Laboratory, University of Oregon, Eugene, Oregon, USA; e Institute for Health in the Built Environment, University of Oregon, Portland, Oregon, USA; f Institute of Ecology & Evolution, University of Oregon, Eugene, Oregon, USA; g Santa Fe Institute, Santa Fe, New Mexico, USA; h Department of Biostatistics, Harvard T. H. Chan School of Public Health, Boston, Massachusetts, USA; i Broad Institute of Massachusetts Institute of Technology and Harvard, Cambridge, Massachusetts, USA; j School of Sustainable Engineering and the Built Environment, Arizona State University, Tempe, Arizona, USA; k School for Biological Health Systems Engineering, Arizona State University, Tempe, Arizona, USA; Pacific Northwest National Laboratory

**Keywords:** antibiotic resistance, microbiome, triclosan

## Abstract

Humans purposefully and inadvertently introduce antimicrobial chemicals into buildings, resulting in widespread compounds, including triclosan, triclocarban, and parabens, in indoor dust. Meanwhile, drug-resistant infections continue to increase, raising concerns that buildings function as reservoirs of, or even select for, resistant microorganisms. Support for these hypotheses is limited largely since data describing relationships between antimicrobials and indoor microbial communities are scant. We combined liquid chromatography-isotope dilution tandem mass spectrometry with metagenomic shotgun sequencing of dust collected from athletic facilities to characterize relationships between indoor antimicrobial chemicals and microbial communities. Elevated levels of triclosan and triclocarban, but not parabens, were associated with distinct indoor microbiomes. Dust of high triclosan content contained increased Gram-positive species with diverse drug resistance capabilities, whose pangenomes were enriched for genes encoding osmotic stress responses, efflux pump regulation, lipid metabolism, and material transport across cell membranes; such triclosan-associated functional shifts have been documented in laboratory cultures but not yet from buildings. Antibiotic-resistant bacterial isolates were cultured from all but one facility, and resistance often increased in buildings with very high triclosan levels, suggesting links between human encounters with viable drug-resistant bacteria and local biocide conditions. This characterization uncovers complex relationships between antimicrobials and indoor microbiomes: some chemicals elicit effects, whereas others may not, and no single functional or resistance factor explained chemical-microbe associations. These results suggest that anthropogenic chemicals impact microbial systems in or around buildings and their occupants, highlighting an emergent need to identify the most important indoor, outdoor, and host-associated sources of antimicrobial chemical-resistome interactions.

**IMPORTANCE** The ubiquitous use of antimicrobial chemicals may have undesired consequences, particularly on microbes in buildings. This study shows that the taxonomy and function of microbes in indoor dust are strongly associated with antimicrobial chemicals—more so than any other feature of the buildings. Moreover, we identified links between antimicrobial chemical concentrations in dust and culturable bacteria that are cross-resistant to three clinically relevant antibiotics. These findings suggest that humans may be influencing the microbial species and genes that are found indoors through the addition and removal of particular antimicrobial chemicals.

## Abstract (precis)

The ubiquitous use of antimicrobial chemicals may have undesired consequences, particularly on microbes in buildings. This study shows that the taxonomy and function of microbes in indoor dust are strongly associated with antimicrobial chemicals—more so than any other feature of the buildings. Moreover, we identified links between antimicrobial chemical concentrations in dust and culturable bacteria that are cross-resistant to three clinically relevant antibiotics. These findings suggest that humans may be influencing the microbial species and genes that are found indoors through the addition and removal of particular antimicrobial chemicals.

## INTRODUCTION

Triclosan, triclocarban, and parabens—most commonly methyl-, ethyl-, propyl-, butyl-, and benzylparabens—are antimicrobial chemicals widely used in tens of thousands of personal care products and consumer materials that leave long-lasting residues on indoor dust. As a result, they are found in most built environments (1, 2). Antimicrobials are typically deposited and transported by humans through use of toothpastes, antiperspirants, detergents, and cosmetics; they are also formulated into cleaning products, paints, flooring, furniture, kitchenware, toys, and appliances to reduce microbial loads in indoor environments (1, 3, 4). Laboratory studies have demonstrated a potential for the proliferation of antimicrobial-resistant microorganisms and genes under exposures to antimicrobial chemicals (4–10), and mathematical models suggest that the role of the built environment in transmission of drug-resistant microorganisms is underestimated (11). Exposures of microorganisms to these chemicals have the potential to drive the propagation of resistances to unrelated antibiotic drugs and biocides (6, 7, 12, 13). Yet, these processes are poorly understood outside the context of pure laboratory cultures (6, 14–16). An understanding of the ways in which widespread and persistent antimicrobial chemicals influence the structure and function of indoor microbial communities in real buildings is needed to manage the emergence and spread of drug-resistant pathogens (17), particularly in buildings like hospitals (18) and athletic facilities (19, 20), where the acquisition of drug-resistant microorganisms is a frequent phenomenon and inputs of anthropogenic chemicals are high.

While the use of antimicrobial chemicals is widespread, relatively little is known about their impacts on environmental microorganisms. Biocides like triclosan and triclocarban inhibit diverse bacteria, but their primary use by consumers is based on a reported effectiveness against Gram-positive species associated with human skin (21, 22) (e.g., Staphylococcus aureus). Triclosan inhibits bacterial growth by targeting the *fabI* acyl carrier protein reductase gene (23), which encodes a key enzyme in fatty acid synthesis. Additional bactericidal effects on species that lack *fabI* have been attributed to impacts on glycolysis or cell membrane function (24, 25). Triclocarban may act similarly (7). Resistance to these biocides has been disproportionately documented in Gram-negative bacterial cultures (15), where putative resistance mechanisms include the overproduction of multidrug efflux pumps (26), cell wall modifications (7, 16), the expression of stress response pathways (9), and genes encoding resistance to unrelated antibiotic drugs (27). Some Gram-positive bacteria exhibit an intrinsic resistance to triclosan because of mutations to the target gene (10, 28) or through the uptake of exogenous lipids to circumvent inhibition of fatty acid synthesis (29, 30). The modes of action and mechanisms of microbial resistance to parabens are less well understood (31). Like triclosan and triclocarban, paraben resistance has been linked to cell wall characteristics and to nonspecific efflux systems (32, 33), triggering concerns over cross-resistance to other antibiotics. Relating anthropogenic biocide and preservative use to indoor microorganisms and their influence on human health has been difficult, due to this broad spectrum of underlying molecular mechanisms (27) and a dearth of relevant studies on whole microbial ecologies *in situ* (2).

To test the association of dust-borne microbiomes with antimicrobial chemicals, we generated what is potentially the first combination of combined molecular profiles of antimicrobial chemicals and microbial communities across multiple occupied buildings. In this study, we combined liquid chromatography-isotope dilution tandem mass spectrometry (LC-ID-MS/MS) with metagenomic shotgun sequencing of vacuum-collected dust from the hallways, offices, and gymnasiums of 42 operating athletic facilities in Oregon, USA (*n* = 116 total samples retained after quality control). Facilities included private fitness clubs, public recreation centers, and studios for dance, yoga, and martial arts and were characterized by variation in building materials, occupancy, and ventilation strategies (see Fig. S1 in the supplemental material). We further leveraged vacuum-collected dust from this set of facilities to evaluate the potential for the emergence of penicillin, macrolide, and tetracycline cross-resistant phenotypes (2) in buildings of various antimicrobial contents by exposing bacterial isolates cultured from dust to antibiotics representative of these classes.

10.1128/mSystems.00200-18.1 FIG S1 Features of the facilities’ occupancy, ventilation, and building material composition. (a to d) The proportions of sampled rooms from each (a) city and (b) space type and with (c) exterior-facing doors and (d) different flooring materials. Features of the facilities’ occupancy, ventilation, and building material composition were quantified using a combination of censuses, member sign-in records, and interviews with facility employees. (e to h) Distributions of (e) person visits day^−1^ m^−2^, (f) the number of functional moisture sources m^−2^ (e.g., water fountains, sinks), (g) the mean number of business hours day^−1^, and (h) the fraction of total business hours with open windows (dimensionless). Download FIG S1, EPS file, 1.3 MB. Copyright © 2018 Fahimipour et al. 2018 Fahimipour et al. This content is distributed under the terms of the Creative Commons Attribution 4.0 International license.

## RESULTS

**FIG 1:**
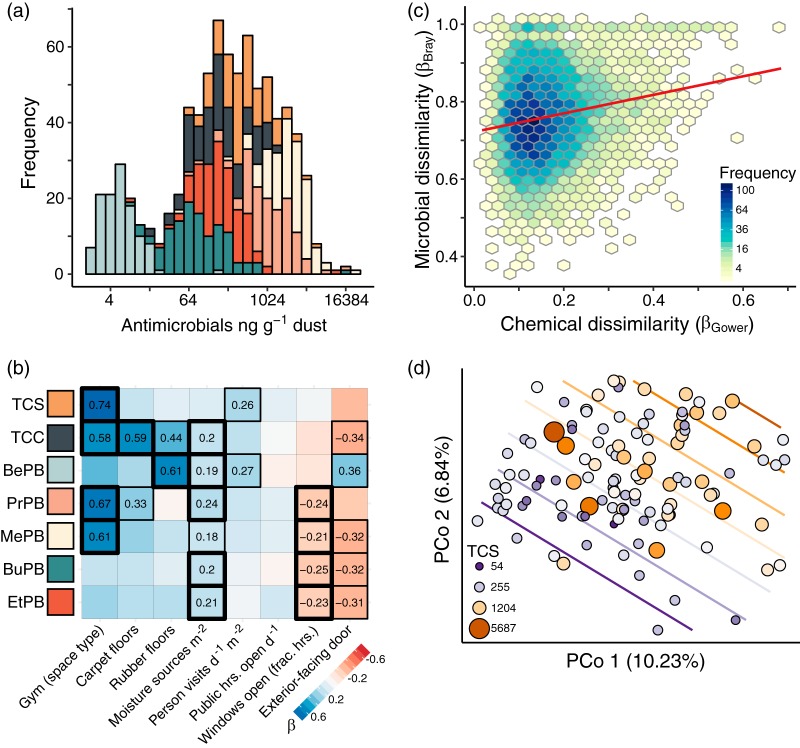
Relationships between antimicrobial chemicals, features of the built environment, and microbial communities. (a) Distributions of triclosan (TCS), triclocarban (TCC), benzylparaben (BePB), propylparaben (PrPB), methylparaben (MePB), butylparaben (BuPB), and ethylparaben (EtPB) concentrations in dust (ng g^−1^) across all sampled athletic facilities (*n* = 116 rooms). (b) Linear correlations between antimicrobial chemicals and building features (results of ANOVA) (Table S1); well-powered building features were retained for analysis using the entropy filter described in Materials and Methods. (c) Chemical profile distance-decay relationship for microbial Bray-Curtis dissimilarities (β_Bray_) and chemical Gower dissimilarities (β_Gower_) between sample pairs. The red line indicates fit from a linear model to raw data. (d) Principal-coordinate analysis (PCoA) visualization of pairwise Bray-Curtis dissimilarities, calculated using Hellinger-transformed species’ relative abundances. Points represent microbial communities from individual rooms, colored and sized by the corresponding triclosan concentration (ng g^−1^ dust). Contour lines show a surface fitted to triclosan values associated with PCoA point coordinates, using generalized additive models as implemented in the R package *vegan*.

Triclosan, triclocarban, and the five assayed parabens were detected in all rooms of all censused facilities at concentrations that were comparable to other indoor environments (34). Total antimicrobial chemical concentrations in dust varied over 4 orders of magnitude (Fig. 1a) and were elevated in gymnasium spaces designated for athletic activities (analysis of variance [ANOVA]; *F*_1,106_ = 2.01, *P = *0.018) and those with more moisture sources per square meter (*F*_1,106_ = 2.12, *P = *0.022); they were marginally elevated in rooms with carpet flooring (*F*_1,106_ = 3.17, *P = *0.072). Methylparaben was most abundant, occurring at a mean concentration (±standard error of the mean [SEM]) of 2,790 ± 256 ng g^−1^ dust, followed by propylparaben (1,528 ± 172 ng g^−1^), triclosan (658 ± 72 ng g^−1^), ethylparaben (352 ± 111 ng g^−1^), triclocarban (300 ± 28 ng g^−1^), butylparaben (258 ± 127 ng g^−1^), and benzylparaben (8 ± 2 ng g^−1^).

10.1128/mSystems.00200-18.4 TABLE S1 Results of ANOVA tests between indoor antimicrobial chemical concentrations and building features. Columns show linear correlation coefficients between centered and scaled antimicrobial chemicals (columns) and building features (rows); well-powered building features were retained for analysis using the entropy filter described in Methods and Materials. Column labels identify the chemicals to which statistics relate. Asterisks mark significance at the *P < *0.05 and *P < *0.01 levels. Download Table S1, PDF file, 0.1 MB. Copyright © 2018 Fahimipour et al. 2018 Fahimipour et al. This content is distributed under the terms of the Creative Commons Attribution 4.0 International license.

Concentrations of individual antimicrobials were also associated with some identifiable building features. Most of the assayed chemicals were individually enriched (ANOVA) (Fig. 1b; see Table S1 in the supplemental material) in spaces designated for athletic activities (compared to hallways or personal offices), in rooms with carpet or rubber mat flooring, and in rooms with more operating water sources per square meter (Fig. 1b; Table S1). Facilities frequented by larger numbers of visitors per day per square meter contained elevated concentrations of triclosan and benzylparaben (Fig. 1b; Table S1). Concentrations of most chemicals were lower in rooms with a direct doorway to the outdoors and in rooms subjected to frequent window ventilation (Fig. 1b; Table S1).

### Dust communities were highly variable and associated with biocides.

Microbial communities in athletic facility dust were highly heterogeneous, comprising a small number of numerically dominant widespread human- and environmentally derived taxa, with many rare species that were specific to individual buildings; only ca. 26% of the detected microbial species (*n* = 370) were observed in more than half of the buildings. Athletic facilities thus lacked a large “core” microbiome. Following the definition of Lloyd-Price et al. (35), species were considered part of the core if they could be detected in more than 75% of buildings. The resulting core comprised six taxa: Propionibacterium acnes, Pseudomonas sp., Massilia sp., C2-like viruses, Subdoligranulum sp., and Enhydrobacter aerosaccus (see Fig. S2a in the supplemental material). Dust largely reflected contributions from occupant skin and urogenital communities (35) (Fig. S2b and c). Despite high species turnover, the composition of phyla did not consistently vary across buildings (permutational multivariate analysis of variance [PERMANOVA]; *R^2^* = 0.013, *P = *0.7) or room types (*R^2^* < 0.001, *P = *0.98) and taxonomically resembled other built environments (36) (Fig. S2d).

10.1128/mSystems.00200-18.2 FIG S2 Distributions and putative origins of microorganisms in athletic facilities. (a) Relationship between building occupancy (the fraction of buildings in which a species was detected) and mean relative abundances (±2 SEM) for species detected at least five times across our data set. A horizontal dashed line marks the occupancy threshold to be considered a member of the “core” microbiome, based on the definition by Lloyd-Price et al. (Nature 550:61–66, 2017, https://doi.org/10.1038/nature23889). Large points above this dashed line comprise species in the athletic facility “core,” which included Propionibacterium acnes, *Pseudomonas* sp., *Massilia* sp., C2-like viruses, *Subdoligranulum* sp., and Enhydrobacter aerosaccus, respectively. Points are colored based on the fraction of subjects in the Expanded Human Microbiome Project (HMP; J. Lloyd-Price et al., Nature 550:61–66, 2017, https://doi.org/10.1038/nature23889) on which they could be detected. A black line shows the fit from a smooth LOESS regression. (b) Meta-analysis of the present buildings, together with subjects from the Expanded HMP using principal-coordinate analysis (PCoA) of species’ relative abundances as described in the main text. Small colored points are microbial communities from HMP subjects colored by body site. Large gray points represent microbial communities from the present set of buildings. (c) Results of microbial source tracking (SourceTracker v1.0; D. Knights et al., Nat Methods 8:761, 2011, https://doi.org/10.1038/nmeth.1650). SourceTracker was trained on HMP samples (b), and predictions were generated by testing the trained model on microbial communities from the present set of buildings. (d) Distribution of phyla across samples; bars represent individual rooms and are ordered based on space type (*x* axis) and the proportions of Proteobacteria, which were the most abundant phylum, on average. Download FIG S2, PDF file, 2.1 MB. Copyright © 2018 Fahimipour et al. 2018 Fahimipour et al. This content is distributed under the terms of the Creative Commons Attribution 4.0 International license.

Antimicrobial chemical concentrations were related to dust community compositions, even when controlling for variation in other factors of each facility’s design, occupancy, and operation. Namely, we calculated predictor matrices for groups of related variables that describe dissimilarity in each space’s (i) antimicrobial chemical profile, (ii) building material census, (iii) human activity, (iv) ventilation strategy, and (v) cleaning product census, together with (vi) a matrix of geographic distances between communities (great circle [km]). These potential drivers of indoor microbial community dissimilarity (see Table S2 and Data Set S1 in the supplemental material), or β-diversity, were used as input to a permutational multiple regression on distance matrix (MRM) analysis (37) to determine the rate of change in microbial β-diversity based on each predictor matrix, with all others held constant. Only dissimilarity in antimicrobial chemical profiles explained a significant amount of variation in dust microbial β-diversity (Fig. 1c) (MRM, partial correlation; *r *=* *0.241, *P = *0.003). Importantly, this relationship held when we accounted for variation in facility design, occupancy, operation, cleaning, and location, suggesting that associations between indoor microbial β-diversity and chemical profiles were not simply recapitulating the effects of covarying building features (e.g., Fig. 1b). A finer-scale analysis revealed that the antimicrobial chemical effect was driven primarily by relationships between the concentrations of triclosan (Fig. 1d) (PERMANOVA; *P = *0.002) and triclocarban (see Fig. S3 in the supplemental material) (*P = *0.013), but not parabens (all *P* values are >0.16), and microbial community species compositions.

10.1128/mSystems.00200-18.3 FIG S3 Relationship between triclocarban levels and dust microbiome composition. Principal-coordinate analysis (PCoA) visualization of pairwise Bray-Curtis dissimilarities, calculated using Hellinger-transformed species relative abundances as described in the main text. Points represent microbial communities from individual rooms, which are colored and sized by the corresponding tricarban (TCC) concentration (ng g^−1^ dust): large pink points represent rooms with high levels, compared to small green points. Contour lines show a surface fitted to triclosan values associated with PCoA point coordinates, using generalized additive models as implemented in the R package *vegan*. Download FIG S3, EPS file, 0.7 MB. Copyright © 2018 Fahimipour et al. 2018 Fahimipour et al. This content is distributed under the terms of the Creative Commons Attribution 4.0 International license.

10.1128/mSystems.00200-18.5 TABLE S2 Groupings of built environment features into dissimilarity matrices. Variable groupings used as input to multiple regression on distance matrix (MRM) analysis. “Dissimilarity category” indicates the group label referred to in the main text. These groups contained sets of variables that are indicated in the “Variable” column; units for these covariates are provided in the “Units” column. Download Table S2, DOCX file, 0.1 MB. Copyright © 2018 Fahimipour et al. 2018 Fahimipour et al. This content is distributed under the terms of the Creative Commons Attribution 4.0 International license.

10.1128/mSystems.00200-18.8 DATA SET S1 Cleaning product ingredient census. Rows represent binary variables representing the presence or absence of chemical ingredients. Values of 1 indicate that the chemical in the row is included in at least one cleaning product routinely used by each facility’s cleaning staff. Columns correspond to individual rooms in each facility. Download Data Set S1, CSV file, 0.01 MB. Copyright © 2018 Fahimipour et al. 2018 Fahimipour et al. This content is distributed under the terms of the Creative Commons Attribution 4.0 International license.

### Biocides were accompanied by an enrichment of taxa with diverse resistance capabilities.

**FIG 2:**
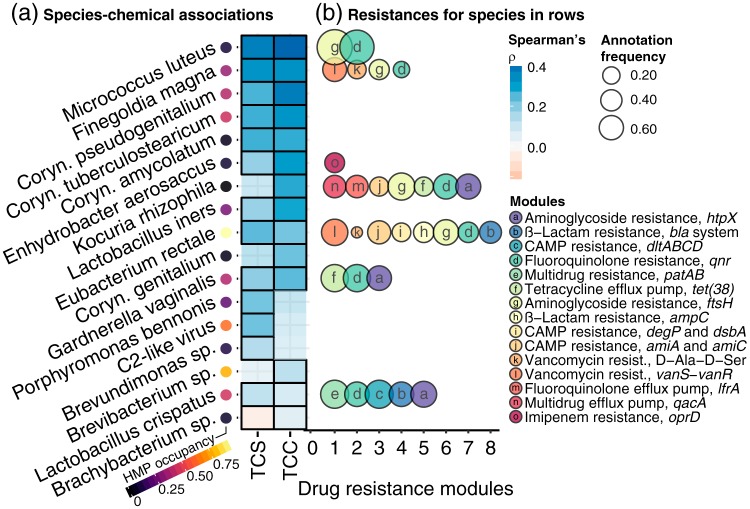
Relationships between biocide concentrations and microbial species. (a) Spearman correlations between microbial species and triclosan (TCS) or triclocarban (TCC) concentrations (ng g^−1^ dust), with significance as determined by HAllA (see Materials and Methods). The margin shows species’ occurrence frequencies for subjects in the Expanded Human Microbiome Project (35). (b) Number of resistance modules annotated in the pangenomes of species in the rows of panel a. Modules are members of the “Drug resistance” and “Drug efflux transporter/pump” KEGG (38) categories. The size of each bubble is scaled proportionally to the fraction of rooms in which both species-specific marker genes (i.e., the results of MetaPhlAn2 [50]) and the drug resistance gene were detected.

Elevated concentrations of the biocides triclosan and triclocarban were most strongly associated with increases in the relative abundances of 17 microbial species (Fig. 2a) by hierarchical all-against-all significance test (HAllA [http://huttenhower.sph.harvard.edu/halla]): 13 Gram-positive or Gram-variable bacterial species, 3 Gram-negative species (Enhydrobacter aerosaccus, Porphyromonas bennonis, and Brevundimonas sp.), and 1 bacteriophage (C2-like virus). A Monte Carlo test indicated that the number of Gram-negative species in this set of antimicrobial associates was less than the null expectation, which was estimated through 10^4^ independent size-biased samples of species from our data set (*P* < 0.001), marking a shift toward Gram-positive-dominated communities in dust with high biocide levels. Antimicrobially enriched species exhibited low and high occurrence frequencies across subjects in the Human Microbiome Project (35) (Fig. 2a), indicating that humans are likely one of multiple sources for biocide tolerance indoors.

The documented variety of biocide resistance mechanisms under laboratory conditions and in other systems suggests a diverse set of resistance strategies occurring in buildings. The pangenomes of several triclosan- and triclocarban-enriched species were annotated with functional KEGG (38) modules that encode resistances to multiple antimicrobial compounds, some of which have conceivable links to biocide tolerance. The most strongly antimicrobially enriched species, Micrococcus luteus, was frequently associated with gyrase-protecting fluoroquinolone resistance, *qnr*, and aminoglycoside resistance through genes that encode membrane protease activity, *ftsH* (Fig. 2b). Cross-resistances between quinolones and other antimicrobial chemicals, including triclosan, have been demonstrated and attributed to upregulated stress responses in Escherichia coli, Salmonella enterica (9), and Pseudomonas aeruginosa (6). The stress response functions associated with FtsH activity have also been linked with resistance to diverse compounds other than aminoglycosides, including β-lactams, and alkaline or saline compounds (39). The *qnr* and *ftsH* modules were also frequently associated with Finegoldia magna, the second most antimicrobially enriched species, as well as Kocuria rhizophila, Eubacterium rectale, Gardnerella vaginalis, and Lactobacillus crispatus (Fig. 2b).

Nonspecific efflux pumps are often a major component of biocide resistance (4–7). Functional modules encoding multidrug efflux pumps could be confidently annotated in the pangenomes of numerous species detected in this set of buildings. A few of these species were potentially among the antimicrobial associates, including the soil-derived species *K. rhizophila* (Fig. 2), which was frequently associated with genes that encode the generic antiseptic efflux protein QacA (40); L. crispatus, associated with the PatAB multidrug efflux protein complex; and *E. rectale*, associated with the envelope protein folding and degrading factors DegP and DsbA, which are involved in the assembly of multidrug transporters (Fig. 2b).

Not all of the species that were enriched in facilities with higher triclosan or triclocarban levels could be associated with drug resistance modules previously implicated with biocide exposures. The triclosan- and triclocarban-enriched taxa Corynebacterium tuberculostearicum, Corynebacterium pseudogenitalium, Corynebacterium amycolatum, and Corynebacterium genitalium were not seen to carry genomic modules involved in drug resistance or efflux (Fig. 2b). Corynebacteria are highly abundant on human skin (41), which can also contain high concentrations of triclosan and triclocarban that are typical in, e.g., antiperspirants (1). The relationship between Corynebacterium and biocide concentrations may exemplify a shared source of chemicals and microorganisms from human perspiration in an athletic setting. Enrichment of C2-like bacteriophages further demonstrates the potential for ecological processes like colonization and interspecific interactions to indirectly drive patterns in these facilities—in this case, changes in the relative abundances or inputs of hosts in the Lactobacillales likely contributed to the observed increase of C2-like phages. Together, these results suggest that the effects of antimicrobials on microbial communities in buildings will be driven by multiple contemporaneous population- and community-level mechanisms, including nonspecific resistances and efflux systems that generate variable chemical tolerances across microbial populations, as is potentially the case for species like M. luteus, F. magna, and K. rhizophila; spatial processes that simultaneously bring both chemicals and new microorganisms into buildings (e.g., the *Corynebacterium* congeners); and ecological dynamics that generate co-occurrence patterns between potentially interacting species (e.g., susceptible or tolerant hosts in the *Lactobacillales* and their phages) in addition to still unidentified functional capabilities.

### Metabolic functions enriched among triclosan associates.

The possibility that microbial functions other than drug resistance or efflux pumps were involved in species’ responses to elevated triclosan or triclocarban concentrations was assessed using a permutational gene set enrichment analysis (GSEA) (42, 43). This analysis identified functional modules that were significantly overrepresented in the pangenomes of species that themselves exhibited stronger biocide associations (based on the results of HAllA [Fig. 2a]) and may provide insight into the functions of microorganisms that are capable of tolerating higher antimicrobial conditions in buildings.

**FIG 3:**
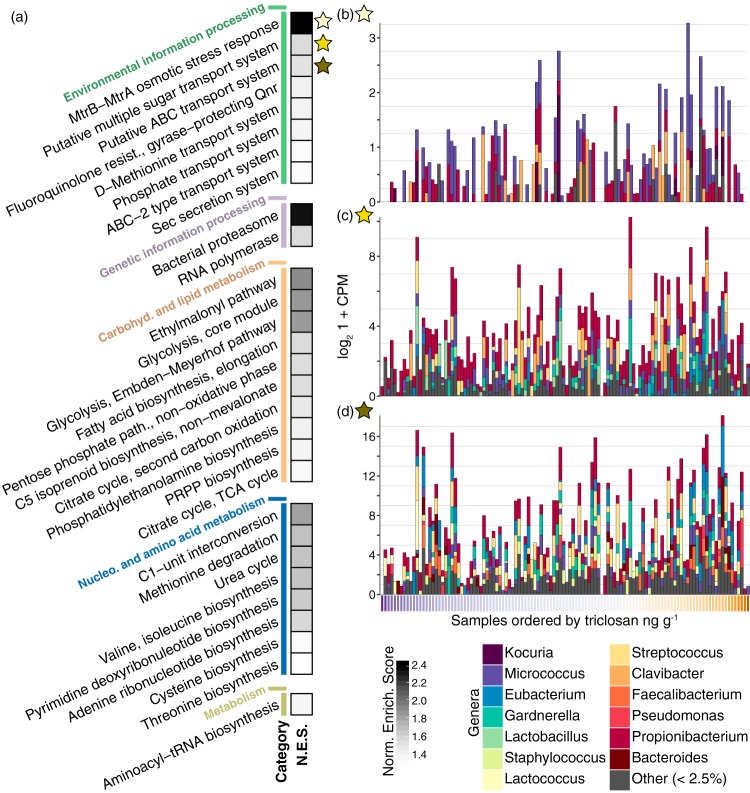
Enrichment of microbial functions with elevated triclosan. (a) Overrepresented functional capabilities among triclosan-related species (results of GSEA [42, 43]). Significantly overrepresented modules are grouped based on KEGG (38) functional categories. (b) Positive relationships between log_2_ 1 + *x*-transformed gene copies per million (CPM) and triclosan levels (ng g^−1^ dust) for the most enriched module, the *mtrAB* transcriptional regulators. Bars represent individual rooms, stratified based on the proportions of species-specific annotations. Marker colors for triclosan levels are the same as in Fig. 1d. (c and d) Bar plots as in panel b for the two functions with the highest positive Spearman rank correlation coefficients with triclosan (ρ = 0.29 and 0.23, respectively).

We identified 29 modules that were overrepresented among triclosan-related species (Fig. 3a; see Table S3 in the supplemental material) and a single module carrying genes involved in glycolysis for triclocarban-related species. For triclosan associates, the most enriched module contained genes encoding the MtrAB complex of transcriptional regulators (Fig. 3a and b; Table S3), which contribute to biocide resistance in bacteria by enhancing transcription of osmotic stress response pathways and nonspecific efflux pumps (44). *mtrAB* belonged to a group of eight enriched modules involved in environmental information processing, which comprised transporters, including sugar, amino acid, phosphate, and ATP-binding cassette (ABC) transporters, and the *sec* bacterial secretion system (Fig. 3a, c, and d; Table S3). This finding is consistent with a recent triclosan addition experiment of anaerobic digesters, which was likewise accompanied by an enrichment of bacterial genes for amino acid, sugar, and ABC transporters (27). It is also consistent with laboratory exposures of Staphylococcus aureus to triclosan that have shown upregulation of ABC transporters (24).

10.1128/mSystems.00200-18.6 TABLE S3 Overrepresented functional capabilities among triclosan-related species. Results of gene set enrichment analysis. Permutation-based *P* values and FDR-adjusted *q* values are shown in the columns labeled “pval” and “qval,” respectively. Significance tests were accomplished through 10^4^ gene set permutations. Columns marked “ES” and “NES” refer to the enrichment score and normalized enrichment score, described by A. Subramanian et al. (Proc Natl Acad Sci U S A 102:15545–15550, 2005, https://doi.org/10.1073/pnas.0506580102). Higher normalized enrichment scores correspond to a greater overrepresentation of molecular functions among the pangenomes of strongly triclosan-enriched species. Download Table S3, DOCX file, 0.1 MB. Copyright © 2018 Fahimipour et al. 2018 Fahimipour et al. This content is distributed under the terms of the Creative Commons Attribution 4.0 International license.

Overrepresentation of molecular functions among triclosan-related species can be explained either because these functions are advantageous under certain conditions, or because of coincidental enrichments of unrelated capabilities that are common or well characterized in this species set, potentially as in the case of essential “housekeeping” genes involved in nucleotide metabolism (Fig. 3a; Table S3). Other enriched metabolic functions had equivocal connections to triclosan, as in the set of 10 functional modules related to carbohydrate and lipid metabolism. This group consisted of genes involved in fatty acid elongation, as well as upstream metabolic processes like glycolysis and the tricarboxylic acid (TCA) cycle (Fig. 3a; Table S3). This result is surprising, as inhibition of fatty acid elongation is perhaps the most-well-studied response of bacterial isolates to triclosan (23, 25). The enrichment of these functions may imply a compensating response to the presence of triclosan or that these pathways are unaffected in triclosan-related species. It is conceivable that widespread resistant isozymes of the *fabI* gene (23) among triclosan associates could explain an enrichment of genes encoding fatty acid elongation, but we found only marginal metagenomic evidence of this possibility (see Table S4 in the supplemental material). Alternatively, abundant exogenous fatty acids have been shown to initiate biochemical feedbacks that allow some Gram-positive bacteria to overcome FabI inhibition in the laboratory (28, 29), and we hypothesize that human-derived fatty acids are abundant in athletic facilities where perspiration is common. Understanding the real-world contexts in which human exudates modulate species’ antimicrobial resistances (28) in dust will be an important direction for future research.

10.1128/mSystems.00200-18.7 TABLE S4 Results of a gene set enrichment analysis for acyl carrier protein reductase gene families. Permutation-based *P* values and FDR-adjusted *q* values are shown in the columns labeled “pval” and “qval,” respectively. Significance tests were accomplished through 10^4^ gene set permutations. Columns marked “ES” and “NES” refer to the enrichment score and normalized enrichment score. “Isozyme” column refers to the acyl carrier protein isozyme name to which the statistics refer. Notably, the triclosan-susceptible *fabI* gene was marginally underrepresented among triclosan-enriched species, although no significant trends emerged from this analysis. Download Table S4, DOCX file, 0.1 MB. Copyright © 2018 Fahimipour et al. 2018 Fahimipour et al. This content is distributed under the terms of the Creative Commons Attribution 4.0 International license.

### Cultivable cross-resistant phenotypes were widespread across facilities.

Our metagenomic analyses detected elevated relative abundances of particular species and functions under high triclosan levels. These species were also associated with cross-resistances toward a diversity of unrelated antibiotic drugs, including β-lactams and tetracyclines (Fig. 2), which were similarly observed by Hartmann et al. (2) in the only other study to our knowledge to combine high-throughput antimicrobial and metagenomic assays in the built environment. We therefore sought to test the possibility that cultivable cross-resistant bacterial phenotypes densities would associate with triclosan concentrations using culture-based antibiotic exposure experiments of *n* = 7,659 colonies isolated from the same vacuum dust used for antimicrobial and metagenomic analyses. Isolates were exposed to each of three clinically important antibacterial drugs–clarithromycin, ampicillin, and tetracycline—representing commonly predicted cross-resistances both herein (Fig. 2b) and in the single building studied by Hartmann et al. (2).

**FIG 4:**
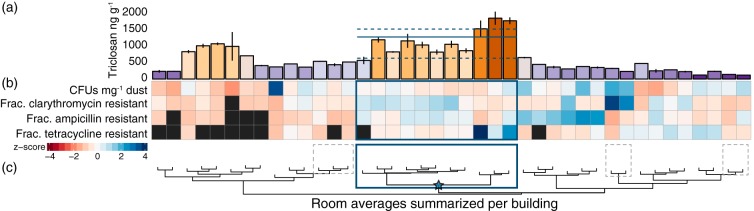
Building groupings based on culture density, diversity, and drug resistance phenotypes. (a) Mean triclosan concentrations (error bars ± 2 SEM) per building level, with colors as in Fig. 1d. Median and quartiles are shown for a large significant cluster of similar buildings (identified in panels b and c). (b) Colony-forming unit (CFU) densities g^−1^ dust and the fractions of CFUs resistant to clarithromycin, ampicillin, and tetracycline. (c) Cluster dendrogram showing Gower dissimilarities between buildings based on features of their culturable communities. Blocks mark clusters with significant support (*P* < 0.01), based on 10^4^ multiscale bootstrap resamples (45) of normalized feature values. The only cluster with significant support that consisted of more than three buildings is marked by a thick blue block and a colored star.

Most colonies were not resistant to any of the applied antibiotics: 71.7% of colonies displayed no resistance to any compounds tested. However, colonies that were resistant to at least one of the assayed drugs could be isolated from all but a single building (Fig. 4), indicating that while relatively few cultivable bacteria were drug resistant, they were widely distributed across facilities.

Hierarchical cluster analysis based on a multiscale bootstrap resampling (45) identified subgroups of buildings with characteristic triclosan levels, CFU densities (CFU mg^−1^ dust), and fractions of colonies with resistances to clarithromycin, ampicillin, or tetracycline (Fig. 4). The largest subgroup comprised 11 buildings (*P* < 0.001) that contained triclosan at levels ca. 3-fold higher than all other buildings on average (Fig. 4) (1,307 ± 209 ng g^−1^ dust compared to 410 ± 32 ng g^−1^ [mean ± SEM]). These buildings were consistently characterized by ca. 3-fold-lower average CFU densities (278.9 ± 39 compared to 827.2 ± 338.4 CFU mg^−1^ dust). Yet colonies isolated from this subgroup of buildings showed the highest proportions of resistance to clarithromycin (27% ± 3% compared to 18% ± 4% in all other colonies), ampicillin (21% ± 2% compared to 16% ± 3%), and tetracycline (5% ± 2% compared to 2 ± 0.4%). Thus, buildings with the very highest triclosan concentrations (typically exceeding 1,000 ng g^−1^ dust) were distinct from all others (Fig. 4). Similar critical transitions in response to triclosan inputs have been documented for microbial genes in anaerobic digester communities (27), and these types of responses may represent a convergence of microbial functions under only the strongest local chemical pressures. The potential for high phenotypic variability across the remaining buildings and small subgroups may reflect more stochastic microbial contributions from human hosts and outdoor environments (46) when chemical pressures are weaker (27).

## DISCUSSION

We provide the first characterization of relationships between common antimicrobial chemicals and indoor microbial community structure and function across multiple occupied buildings. A limitation of this study is that we were unable to determine whether these relationships (Fig. 2 and 3) reflected processes occurring inside or outside buildings—for instance in tap water (1), soils, freshwater habitats (47), or on human hosts (8). Nevertheless, results consistently pointed to effects of triclosan in particular on the indoor microbiome (Fig. 1c and d) and a diversity of potentially related functional responses (Fig. 2 and 3) to these chemical pressures, including changes in the numbers of genes for efflux systems, outer membrane characteristics, osmotic stress responses, material transporters, and aspects of lipid metabolism. At a minimum, our results suggest that anthropogenic antimicrobial chemicals and microbial systems are interacting somewhere in or around these buildings or their occupants; this is reflected in the time-integrated chemical and biological profiles of indoor dust. Moreover, the observation that human occupancy could not explain that these alternate possibilities are not mutually exclusive and are likely taxon or gene specific raises the need to identify salient indoor, outdoor, and human-associated sites of antimicrobial chemical-resistome interactions. This knowledge will be essential in efforts to design targeted chemical or microbial interventions aimed at limiting the spread of antibiotic drug-resistant microorganisms. At present, it is unclear where these efforts ought to be focused.

Bacterial genes encoding transporters have now been associated with triclosan in multiple ecosystem types (Fig. 3) (27), yet the precise roles and relative contributions for many of these functions in microbial biocide tolerance remain uncertain. Research that extends our approach by applying metatranscriptomic techniques to dust communities (48) will be effective for testing the generality of our results and for parsing relationships that manifest indoors versus outdoors. Additional studies are also needed to document factors that modulate rates of human colonization by both viable drug-resistant bacteria and relic resistance genes from the built environment. The present study represents an important early step in a long-term research plan, and future work is still needed to accept or reject the hypothesis that triclosan impacts microbial communities *in situ*, independent of other architectural and historical contingencies. As this study was conducted just prior to the 2017 federal ban on triclosan in soaps, these data serve as a critical reference point for future work.

Culture-based antibiotic exposure experiments revealed links between triclosan concentrations in dust and the culturable densities of viable and cross-resistant bacterial phenotypes for three clinically relevant antibiotics. A challenge for future work will be to define triclosan resistance thresholds for diverse environmental microbial isolates (i.e., MICs) and to develop robust, reproducible methods for testing triclosan resistances in thousands of isolates with a large variety of potential genomic or metabolic functions underlying resistances to the same chemical. The development of high-throughput methodologies will enable screening for triclosan resistance phenotypes, which in turn will enable the elucidation of any cross-resistance mechanisms in isolates that are driven by triclosan exposures.

### Conclusions.

The problem of antimicrobial chemical dissemination in buildings is complicated by the fact that built environments are complex systems representing a mixture of microorganisms that arrive from disparate hosts and habitats, each responding to chemical conditions and contributing to the abundances of indoor antimicrobial resistance genes in different ways. A motivation behind the recent federal triclosan ban in hand soaps was to help curb this problem by slowing chemical inputs to the built environment (47). Yet these chemicals leave long-lasting residues and are still widely used in cosmetics, plastics, and building materials (1), emphasizing the urgent need to document chemical drivers of indoor resistome dynamics (49) and how they ultimately relate to the transmission of latent and infectious drug-resistant pathogens to humans.

## MATERIALS AND METHODS

We studied relationships between antimicrobial chemicals and microbial communities across multiple occupied athletic facilities using a combination of liquid chromatography-isotope dilution tandem mass spectrometry (LC-ID-MS/MS) and metagenomic shotgun sequencing of vacuum-collected dust from 42 operating athletic facilities in Oregon, USA. Facilities included private fitness clubs, public recreation centers, and studios for dance, yoga, and martial arts. Features of the facilities’ occupancy, ventilation, and building material composition were quantified using a combination of censuses, member sign-in or count records, and interviews with facility employees. These data included room areas in square meters, space types (i.e., whether spaces were used for athletic activities, as hallways, or as personal offices), the number of functional moisture sources per square meter (e.g., water fountains, sinks), the mean number of business hours per day, the mean number of person visits per day per square meter, the fraction of business hours with open windows, the use of mechanical ventilation, the presence of exterior-facing doors, and a census of flooring and wall materials.

### Sample collection, DNA extraction, and metagenomic sequencing.

Dust was collected at each site using a vacuum fitted with Dustream collectors (Indoor Biotechnologies, Charlottesville, VA). Collector filters (40-μm-pore mesh) containing the vacuumed dust were placed into sterile Nasco Whirl-Paks bags (Nasco, Fort Atkinson, WI) and stored under dark conditions at room temperature during collections. Collection took place from 18 July to 21 November 2016. Each sample was homogenized, and 0.25 g of dust was aliquoted into sterile 2-ml tubes and stored at −80°C until DNA extraction.

The DNA from dust aliquots was extracted using the MoBio PowerLyzer PowerSoil DNA isolation kit (MoBio, Carlsbad, CA, USA) protocol. To perform a metagenomic analysis, 1 ng of gDNA from each sample was prepared using the Illumina Nextera XT DNA library prep kit, along with the corresponding Illumina index kits v2 set A and set B, following the manufacturer’s instructions through the amplification step. Amplified products were purified with a modified bead-based DNA cleanup protocol using Mag-Bind RxnPure Plus by Omega Bio-Tek (Norcross, GA), quantified using the Quant-iT double-stranded DNA (dsDNA) assay kit, and pooled with equal concentrations of product using an Eppendorf (Hamburg, DE) epMotion 5075 robot. Libraries were sequenced on an Illumina HiSeq 4000 with 150-bp paired-end reads (insert size ranged from 250 to 1,000 bp).

### Chemical extraction and quantification.

The remaining homogenized dust from each collection was aliquoted in duplicates or triplicates—depending on how much dust was collected—of ca. 0.1 g each and analyzed for antimicrobial compounds using a modified dispersive solid-phase extraction (d-SPE) followed by LC-ID-MS/MS, as described in detail by Chen et al. (34). We measured concentrations (ng g^−1^ dust) of triclosan, triclocarban, and methyl-, propyl-, ethyl-, butyl-, and benzylparabens, which prior research has indicated are ubiquitous in indoor dust (1, 2, 34).

### Bacterial cultivation and isolation.

Approximately 20 mg of dust was weighed and suspended in 50 ml of suspension buffer containing 42.5 mg liter^−1^ KH_2_PO_4_ (Fisher BioReagents, Pittsburgh, PA), 250 mg liter^−1^ MgSO_4_·7H_2_O (Sigma-Aldrich, St. Louis, MO), 8.0 mg liter^−1^ NaOH (MP Biomedicals, Solon, OH), and 0.02% (vol/vol) Tween 80 (Sigma-Aldrich, St. Louis, MO) in deionized water. Each dust suspension was shaken on an orbital shaker (compact mini digital rotator; Thermo Scientific, Waltham, MA) for 10 min. After shaking, 100 µl of suspension was spread onto Trypticase soy agar (TSA) supplemented with 4 µg ml^−1^ itraconazale (Alfa Aesar, Ward Hill, MA) in triplicate and grown aerobically at 25°C for up to 4 days. After growth, colonies were counted and identified based on seven morphological characteristics.

### Replica plating and antibiotic screening.

Master TSA plates were replicated to TSA plates containing 4 µg ml^−1^ itraconazale and one of the following antibiotic agents: 100 µg ml^−1^ ampicillin (Acros Organics, Geel, Belgium), 2.0 µg ml^−1^ clarithromycin (Tokyo Chemical Industry, Co., Tokyo, Japan), or 10.0 µg ml^−1^ tetracycline (Chem-Impex International, Inc., Wood Dale, IL). Isolates were replica plated using velveteen squares for transfer from the master plate to ampicillin, clarithromycin, and tetracycline plates. Plates were then incubated aerobically at 25°C for up to 4 days. Antibiotic-supplemented plates were then compared to the master plate, and each of the bacterial isolates was recorded as resistant or sensitive. Results were aggregated at the building level for analyses.

### Metagenomic data processing.

Forward reads for each sample were filtered and trimmed, and low-quality reads, human sequences, sequences present in the negative extraction kit, and PCR controls were removed using KneadData v0.6.1 (http://huttenhower.sph.harvard.edu/kneaddata) with default parameter settings and the “Homo_sapiens_db” reference database. Taxonomic compositions and abundances were determined from trimmed and filtered reads using MetaPhlAn2 (50) v2.7.7 with default settings. Microbial functional potential, including functions involved in drug resistance, were quantified using HUMAnN2 (35, 51) v0.11.1 with KEGG (Kyoto Encyclopedia of Genes and Genomes [38]) gene family annotations. Briefly, HUMAnN2 constructs a reference database from the pangenomes of species detected in each sample by MetaPhlAn2 and maps reads against this reference to quantify gene relative abundances (copies per million [CPM]) on a per-species basis. The remaining unmapped reads are mapped by search against a UniRef-based database (52). Gene family annotations made using the KEGG reference database were parsed into modules using KEGG mapper (38) and HUMAnN2 for analysis.

### Statistical analyses.

Analyses were performed using the statistical programming environment R and hierarchical all-against-all significance testing (HAllA v0.7.18 [http://huttenhower.sph.harvard.edu/halla]). Associations between log_2_-transformed antimicrobial chemical concentrations and measured features of built environment design and operation were quantified using ANOVA. We fit linear models of the form *y* = β_1_(*x*_1_) + β_2_(*x*_2_) + … β*_n_*(*x_n_*) + *E*, where *y* is the log_2_-transformed concentration of an antimicrobial chemical (ng g^−1^ dust), β*_i_* values are linear regression coefficients for fixed effects *x_i_*, and *E* is a vector of errors. Normality of residuals was confirmed for models using quantile-quantile plots. We report standardized effects that reflect the per-unit relationship between centered and scaled log_2_ 1 + *x*-transformed predictors and antimicrobial chemical concentrations.

Microbial community dissimilarities or β-diversities were quantified as pairwise Bray-Curtis distances, calculated using Hellinger-transformed species’ relative abundances (i.e., the output of MetaPhlAn2). Dissimilarities for the five predictor distance matrices (see Table S2 for a description of measured building features) were calculated using the Gower metric because of its improved performance (53) with mixed data types. These matrices were used as input for a permutational multiple regression on distance matrix (MRM) analysis (37) to determine the rate of change in β-diversity as a function of dissimilarity in predictor matrices. The effects of antimicrobial chemicals on the community compositions of dust were quantified using a permutational multivariate analysis of variance (PERMANOVA) with 10^4^ matrix permutations. A permutational hierarchical all-against-all significance test (HAllA v0.7.18; http://huttenhower.sph.harvard.edu/halla) was used to detect species that were significantly Spearman rank correlated with triclosan and triclocarban concentrations, while controlling for type I error. We limited analyses to species that were sufficiently powered using HAllA’s default entropy filter based on a discretization by principal-component analysis. *P* values were calculated using 10^4^ matrix permutations and adjusted to *q* values using the Benjamini-Hochberg false-discovery rate (FDR) procedure (54). Associations with *P < *0.01 and *q *<* *0.1 were considered significant.

GSEA (42) using preranked species sets (i.e., the results of HAllA) was accomplished using the fast gene set enrichment package *fgsea* (43) in R. Functional modules with sufficient power to include in the GSEA were selected using the univariate entropy filter described above. Species’ association ranks with triclosan and triclocarban were calculated as *r_k_* = sgn(ρ*_k_*) × 1/*p_k_*, where *r_k_* is the rank of species *k* used for analysis, ρ*_k_* is the Spearman rank similarity coefficient calculated by HAllA, and *p_k_* is the associated permutation-based *P* value. Functional modules were assigned to species’ gene sets if both species-specific marker genes (i.e., the results of MetaPhlAn2) and the module were detected together at least once. Enrichments with *P < *0.01 and FDR-adjusted (54) *q *<* *0.1 were retained as significant.

Hierarchical clustering based on feature of facilities’ culturable communities was conducted with a Ward linkage method using Gower distances. Cluster significance was calculated using 10^3^ multiscale bootstrap resamples as implemented in the *pvclust* package (45), with a threshold of *P < *0.01 indicating that clusters were well supported.

### Accession number(s).

The sequence files generated during the present study are available in the Sequence Read Archive (SRA) under accession no. PRJNA489265.
