# Supplementary material for: Antimicrobial Chemicals Associate with Microbial Function and Antibiotic Resistance Indoors
Source: mSystems. 2018 Dec 11;3(6):e00200-18. doi: 10.1128/mSystems.00200-18 (PMC6290264; doi:10.1128/mSystems.00200-18)
Supplement: TABLE S1 [file sys006182300st1.pdf]

Statistical results: Antibiotic chemicals

Table 1:

	$\log_2$ TCC	$\log_2$ TCS	$\log_2$ MePB	$\log_2$ PrPB	$\log_2$ EtPB	$\log_2$ BuPB	$\log_2$ BePB
Gym (space type)	0.575**	0.744**	0.606**	0.671**	0.291	0.191	0.417
	(0.281)	(0.299)	(0.286)	(0.281)	(0.290)	(0.293)	(0.292)
Moisture sources m^{-2}	0.202**	0.106	0.178*	0.237***	0.212**	0.199**	0.192**
	(0.090)	(0.096)	(0.091)	(0.090)	(0.093)	(0.094)	(0.093)
Windows open (frac. bus. hrs.)	−0.132	0.024	−0.211**	−0.240**	−0.228**	−0.248**	−0.111
	(0.099)	(0.105)	(0.101)	(0.099)	(0.102)	(0.103)	(0.103)
Public hrs. day^{-1}	0.005	0.084	0.094	0.093	0.147	−0.026	−0.054
	(0.093)	(0.099)	(0.095)	(0.093)	(0.096)	(0.097)	(0.097)
Visits day^{-1} m^{-2}	0.214	0.258*	0.147	0.171	0.031	0.049	0.274*
	(0.140)	(0.149)	(0.142)	(0.140)	(0.144)	(0.145)	(0.145)
Carpet floor	0.588***	0.189	0.305	0.326*	0.227	0.187	0.264
	(0.188)	(0.200)	(0.191)	(0.188)	(0.194)	(0.196)	(0.195)
Rubber floor	0.435*	0.092	0.194	−0.025	0.232	0.096	0.608**
	(0.224)	(0.239)	(0.228)	(0.225)	(0.232)	(0.234)	(0.233)
Exterior-facing door	−0.337*	−0.297	−0.324*	−0.244	−0.314*	−0.318*	0.355*
	(0.182)	(0.194)	(0.185)	(0.182)	(0.188)	(0.189)	(0.189)
Adjusted R^2	0.155	0.040	0.125	0.153	0.100	0.083	0.088
F Statistic (df = 8; 106)	3.614***	1.587	3.042***	3.566***	2.587**	2.282**	2.376**

Note: *p<0.1; **p<0.05; ***p<0.01
