# Supplementary figures and images for: Antimicrobial Chemicals Associate with Microbial Function and Antibiotic Resistance Indoors
Source: mSystems. 2018 Dec 11;3(6):e00200-18. doi: 10.1128/mSystems.00200-18 (PMC6290264; doi:10.1128/mSystems.00200-18)

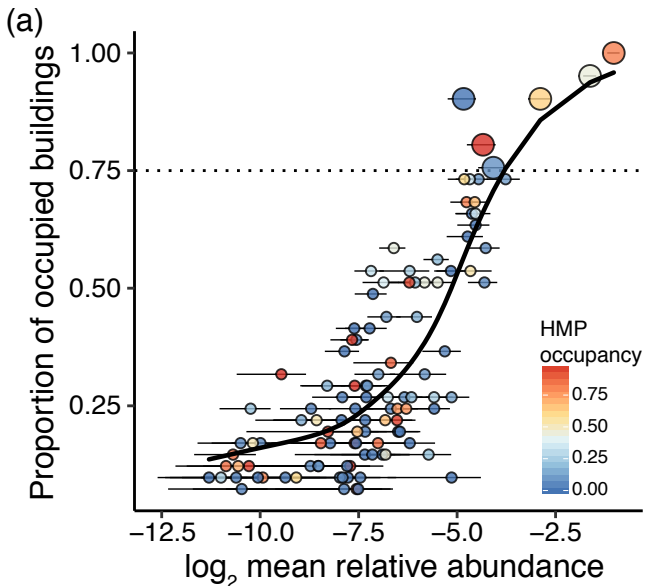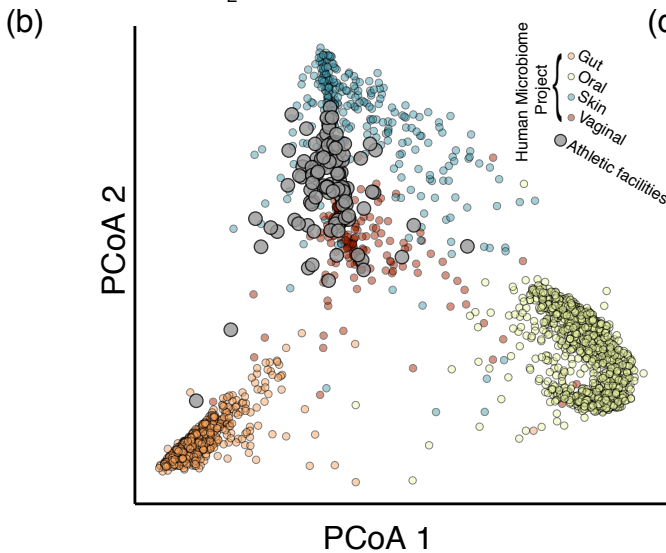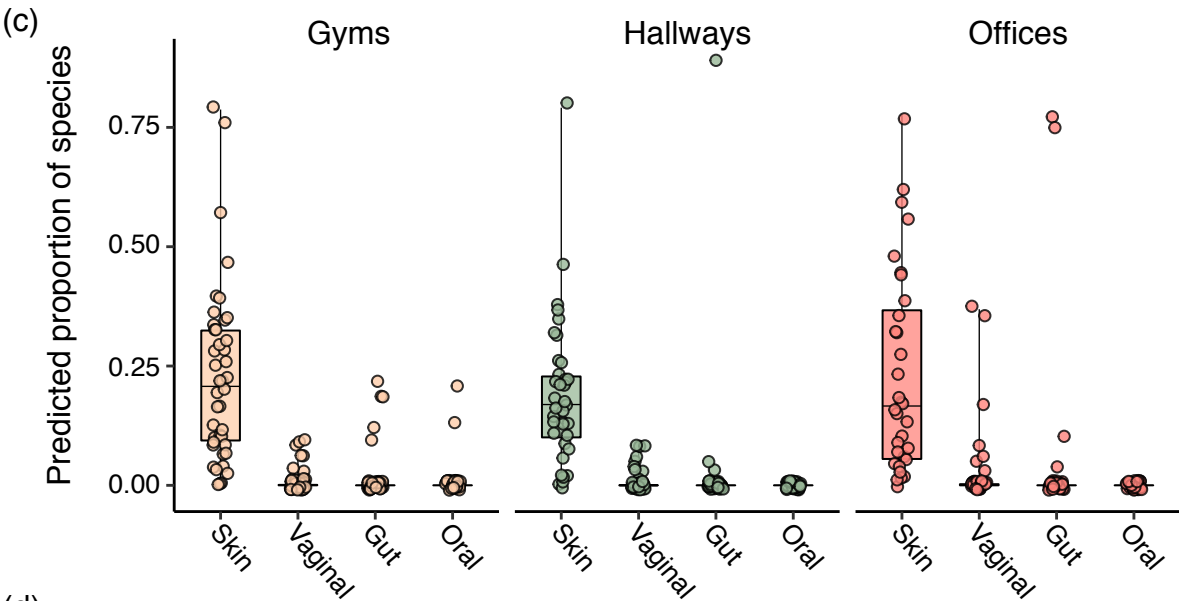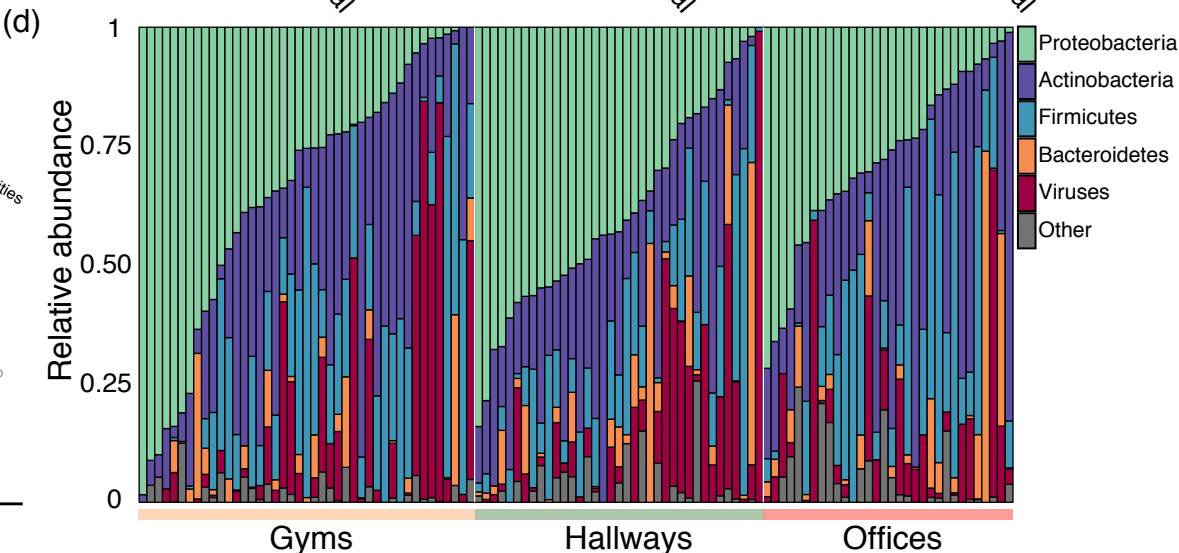

Supplement: FIG S2 [file sys006182300sf2.pdf]
