# Supplementary material for: Antimicrobial Chemicals Associate with Microbial Function and Antibiotic Resistance Indoors
Source: mSystems. 2018 Dec 11;3(6):e00200-18. doi: 10.1128/mSystems.00200-18 (PMC6290264; doi:10.1128/mSystems.00200-18)
Supplement: TABLE S2 [file sys006182300st2.docx]

| **Dissimilarity category** | **Variable** | **Units** |
| --- | --- | --- |
| Antimicrobial chemical profile |  |  |
|  | Triclosan | ng/g dust |
|  | Triclocarban | ng/g dust |
|  | Methylparaben | ng/g dust |
|  | Ethylparaben | ng/g dust |
|  | Butylparaben | ng/g dust |
|  | Benzylparaben | ng/g dust |
|  | Propylparaben | ng/g dust |
| Building materials census |  |  |
|  | Carpet floor | binary variable |
|  | Rubber mat floor | binary variable |
|  | Ceramic tile floor | binary variable |
|  | Concrete floor | binary variable |
|  | Linoleum floor | binary variable |
|  | Wood floor | binary variable |
|  | Brick wall | binary variable |
|  | Concrete wall | binary variable |
|  | Painted drywall wall | binary variable |
|  | Glass wall | binary variable |
| Human activity |  |  |
|  | Person visits | visitors/day m^2^ |
|  | Public hours open | hours/day |
|  | Operating moisture sources | sources/m^2^ |
|  | Gym (space type) | binary variable |
| Ventilation strategy |  |  |
|  | Mechanical ventilation | binary variable |
|  | Fraction of hours window ventilated | dimensionless |
