# Supplementary material for: Antimicrobial Chemicals Associate with Microbial Function and Antibiotic Resistance Indoors
Source: mSystems. 2018 Dec 11;3(6):e00200-18. doi: 10.1128/mSystems.00200-18 (PMC6290264; doi:10.1128/mSystems.00200-18)
Supplement: TABLE S3 [file sys006182300st3.docx]

| **module** | **pval** | **qval** | **ES** | **NES** |
| --- | --- | --- | --- | --- |
| M00461\|MtrB-MtrA (osmotic stress response) two-component regulatory system | 0.002174701 | 0.011494848 | 0.701791292 | 2.443696942 |
| M00342\|Bacterial proteasome | 0.005545287 | 0.024621072 | 0.66569829 | 2.342355327 |
| M00443\|SenX3-RegX3 (phosphate starvation response) two-component regulatory system | 0.019934759 | 0.059838275 | 0.653534321 | 2.275662064 |
| M00373\|Ethylmalonyl pathway | 0.00997921 | 0.037754588 | 0.520112916 | 1.889451106 |
| M00032\|Lysine degradation, lysine => saccharopine => acetoacetyl-CoA | 0.010462777 | 0.037754588 | 0.503854165 | 1.8672624 |
| M00002\|Glycolysis, core module involving three-carbon compounds | 0.000303767 | 0.002633702 | 0.376949476 | 1.837366219 |
| M00088\|Ketone body biosynthesis, acetyl-CoA => acetoacetate/3-hydroxybutyrate/acetone | 0.017706237 | 0.056154067 | 0.495200787 | 1.835193345 |
| M00001\|Glycolysis (Embden-Meyerhof pathway), glucose => pyruvate | 0.000287853 | 0.002633702 | 0.360321841 | 1.823370495 |
| M00140\|C1-unit interconversion, prokaryotes | 0.000242542 | 0.002633702 | 0.307753489 | 1.797769684 |
| M00491\|arabinogalactan oligomer/maltooligosaccharide transport system | 0.013832384 | 0.047981082 | 0.455733929 | 1.717206545 |
| M00035\|Methionine degradation | 0.000245339 | 0.002633702 | 0.292310196 | 1.68583115 |
| M00029\|Urea cycle | 0.00026462 | 0.002633702 | 0.309113129 | 1.675686555 |
| M00019\|Valine/isoleucine biosynthesis, pyruvate => valine / 2-oxobutanoate => isoleucine | 0.000254907 | 0.002633702 | 0.299775088 | 1.673781404 |
| M00533\|Homoprotocatechuate degradation, homoprotocatechuate => 2-oxohept-3-enedioate | 0.314355429 | 0.459124377 | 0.452165789 | 1.655622089 |
| M00053\|Pyrimidine deoxyribonuleotide biosynthesis, CDP/CTP => dCDP/dCTP,dTDP/dTTP | 0.000214731 | 0.002633702 | 0.240069934 | 1.631869135 |
| M00049\|Adenine ribonucleotide biosynthesis, IMP => ADP,ATP | 0.000215378 | 0.002633702 | 0.236097804 | 1.576382382 |
| M00183\|RNA polymerase, bacteria | 0.00021254 | 0.002633702 | 0.218346877 | 1.570763851 |
| M00083\|Fatty acid biosynthesis, elongation | 0.000239177 | 0.002633702 | 0.264159696 | 1.564927624 |
| M00207\|Putative multiple sugar transport system | 0.001091306 | 0.006915242 | 0.370723883 | 1.56350483 |
| M00007\|Pentose phosphate pathway, non-oxidative phase, fructose 6P => ribose 5P | 0.000308452 | 0.002633702 | 0.323143311 | 1.559481818 |
| M00096\|C5 isoprenoid biosynthesis, non-mevalonate pathway | 0.000222568 | 0.002633702 | 0.243706626 | 1.553058706 |
| M00258\|Putative ABC transport system | 0.00024888 | 0.002633702 | 0.267520032 | 1.524385251 |
| M00526\|Lysine biosynthesis, DAP dehydrogenase pathway, aspartate => lysine | 0.08716707 | 0.193510896 | 0.396506004 | 1.516728618 |
| M00011\|Citrate cycle, second carbon oxidation, 2-oxoglutarate => oxaloacetate | 0.00052233 | 0.003865239 | 0.271623664 | 1.487456182 |
| M00155\|Cytochrome c oxidase, prokaryotes | 0.230110159 | 0.381227279 | 0.384209884 | 1.452870749 |
| M00729\|Fluoroquinolone resistance, gyrase-protecting protein Qnr | 0.00052233 | 0.003865239 | 0.261651023 | 1.432844346 |
| M00093\|Phosphatidylethanolamine (PE) biosynthesis, PA => PS => PE | 0.001078167 | 0.006915242 | 0.266201336 | 1.427363548 |
| M00238\|D-Methionine transport system | 0.001121391 | 0.006915242 | 0.274270082 | 1.418512042 |
| M00005\|PRPP biosynthesis, ribose 5P => PRPP | 0.001195886 | 0.006986493 | 0.238112151 | 1.410617474 |
| M00222\|Phosphate transport system | 0.0018917 | 0.010498936 | 0.233750375 | 1.39832137 |
| M00360\|Aminoacyl-tRNA biosynthesis, prokaryotes | 0.003386243 | 0.017085137 | 0.196471123 | 1.396016605 |
| M00009\|Citrate cycle (TCA cycle, Krebs cycle) | 0.008520791 | 0.035029917 | 0.312295698 | 1.379022239 |
| M00021\|Cysteine biosynthesis, serine => cysteine | 0.003742084 | 0.018059623 | 0.269175369 | 1.362133434 |
| M00254\|ABC-2 type transport system | 0.004911811 | 0.022717124 | 0.21454186 | 1.361334339 |
| M00335\|Sec (secretion) system | 0.009184109 | 0.036408434 | 0.19325883 | 1.349256023 |
| M00157\|F-type ATPase, prokaryotes and chloroplasts | 0.010544074 | 0.037754588 | 0.181740746 | 1.338206021 |
| M00018\|Threonine biosynthesis, aspartate => homoserine => threonine | 0.008355795 | 0.035029917 | 0.246368624 | 1.321021143 |
| M00362\|Nucleotide sugar biosynthesis, prokaryotes | 0.015831135 | 0.051683998 | 0.219707095 | 1.296216148 |
| M00570\|Isoleucine biosynthesis, threonine => 2-oxobutanoate => isoleucine | 0.014798094 | 0.049775406 | 0.228064904 | 1.29390321 |
| M00240\|Iron complex transport system | 0.019946092 | 0.059838275 | 0.238971843 | 1.281359826 |
| M00003\|Gluconeogenesis, oxaloacetate => fructose-6P | 0.025853659 | 0.07358349 | 0.219221088 | 1.272802227 |
| M00742\|Aminoglycoside resistance, protease FtsH | 0.057502552 | 0.138451527 | 0.284304346 | 1.258849238 |
| M00535\|Isoleucine biosynthesis, pyruvate => 2-oxobutanoate | 0.046757164 | 0.123572506 | 0.255779961 | 1.25310229 |
| M00209\|Osmoprotectant transport system | 0.182457439 | 0.329392048 | 0.298893031 | 1.245846294 |
| M00572\|Pimeloyl-ACP biosynthesis, BioC-BioH pathway, malonyl-ACP => pimeloyl-ACP | 0.053736875 | 0.135563479 | 0.258849438 | 1.242916352 |
| M00052\|Pyrimidine ribonucleotide biosynthesis, UMP => UDP/UTP,CDP/CTP | 0.039613082 | 0.109926301 | 0.201866727 | 1.239358263 |
| M00236\|Putative polar amino acid transport system | 0.058623619 | 0.138451527 | 0.240645421 | 1.231355722 |
| M00149\|Succinate dehydrogenase, prokaryotes | 0.351217656 | 0.506300777 | 0.303637474 | 1.230222661 |
| M00632\|Galactose degradation, Leloir pathway, galactose => alpha-D-glucose-1P | 0.064823642 | 0.149904671 | 0.261648884 | 1.229823911 |
| M00051\|Uridine monophosphate biosynthesis, glutamine (+ PRPP) => UMP | 0.056579511 | 0.138451527 | 0.17800271 | 1.219218035 |
| M00020\|Serine biosynthesis, glycerate-3P => serine | 0.096377749 | 0.205729426 | 0.262921147 | 1.21652573 |
| M00045\|Histidine degradation, histidine => N-formiminoglutamate => glutamate | 0.146896328 | 0.294960164 | 0.258237367 | 1.188644304 |
| M00127\|Thiamine biosynthesis, AIR => thiamine-P/thiamine-2P | 0.09541779 | 0.205729426 | 0.220340425 | 1.181458721 |
| M00190\|Iron(III) transport system | 0.622291022 | 0.750807646 | 0.293031929 | 1.167958362 |
| M00549\|Nucleotide sugar biosynthesis, glucose => UDP-glucose | 0.195009849 | 0.338220207 | 0.255532562 | 1.166905635 |
| M00114\|Ascorbate biosynthesis, plants, glucose-6P => ascorbate | 0.183984747 | 0.329392048 | 0.245366588 | 1.153292507 |
| M00196\|Multiple sugar transport system | 0.423500612 | 0.572687648 | 0.310115679 | 1.150658972 |
| M00022\|Shikimate pathway, phosphoenolpyruvate + erythrose-4P => chorismate | 0.155688623 | 0.303183107 | 0.185368799 | 1.13806944 |
| M00432\|Leucine biosynthesis, 2-oxoisovalerate => 2-oxoisocaproate | 0.18353301 | 0.329392048 | 0.20162202 | 1.125747943 |
| M00028\|Ornithine biosynthesis, glutamate => ornithine | 0.240947075 | 0.393310667 | 0.213794547 | 1.113461425 |
| M00307\|Pyruvate oxidation, pyruvate => acetyl-CoA | 0.267145852 | 0.406208076 | 0.225611652 | 1.110674588 |
| M00336\|Twin-arginine translocation (Tat) system | 0.485478666 | 0.633978023 | 0.260707348 | 1.108331488 |
| M00176\|Assimilatory sulfate reduction, sulfate => H2S | 0.414039649 | 0.572687648 | 0.235213896 | 1.082669254 |
| M00119\|Pantothenate biosynthesis, valine/L-aspartate => pantothenate | 0.376786735 | 0.536196508 | 0.210599079 | 1.070706706 |
| M00027\|GABA (gamma-Aminobutyrate) shunt | 0.720456116 | 0.800870941 | 0.236345524 | 1.029204323 |
| M00582\|Energy-coupling factor transport system | 0.854786546 | 0.895106666 | 0.168333244 | 0.778871249 |
| M00458\|ResE-ResD (aerobic and anaerobic respiration) two-component regulatory system | 0.602288022 | 0.735163525 | 0.242453915 | 0.778182165 |
| M00298\|Multidrug/hemolysin transport system | 0.561071429 | 0.707715097 | 0.218801627 | 0.75019967 |
| M00228\|Putative glutamine transport system | 0.73605806 | 0.801004359 | 0.167526322 | 0.595467152 |
| M00189\|Molybdate transport system | 0.781284404 | 0.833870854 | 0.139843218 | 0.588049024 |
| M00454\|KdpD-KdpE (potassium transport) two-component regulatory system | 0.673764564 | 0.774941425 | 0.149139585 | 0.574692258 |
| M00256\|Cell division transport system | 0.847270214 | 0.895106666 | 0.127872647 | 0.556841859 |
| M00627\|beta-Lactam resistance, Bla system | 0.923817161 | 0.949478749 | 0.120079028 | 0.440728522 |
| M00842\|Tetrahydrobiopterin biosynthesis, GTP => BH4 | 0.990869393 | 0.990869393 | -0.09578682 | -0.444711614 |
| M00495\|AgrC-AgrA (exoprotein synthesis) two-component regulatory system | 0.975226425 | 0.98409212 | -0.115769555 | -0.508775641 |
| M00299\|Spermidine/putrescine transport system | 0.947614328 | 0.965001747 | -0.090784398 | -0.545703214 |
| M00279\|PTS system, galactitol-specific II component | 0.91382739 | 0.947989161 | -0.137401997 | -0.613073851 |
| M00439\|Oligopeptide transport system | 0.753957879 | 0.812517715 | -0.125710221 | -0.769014986 |
| M00275\|PTS system, cellobiose-specific II component | 0.726568758 | 0.800870941 | -0.146594667 | -0.789317617 |
| M00394\|RNA degradosome | 0.728720406 | 0.800870941 | -0.132611128 | -0.792477698 |
| M00211\|Putative ABC transport system | 0.68418252 | 0.774941425 | -0.175577694 | -0.822536762 |
| M00766\|Streptomycin resistance, deactivating enzyme StrAB | 0.684167462 | 0.774941425 | -0.211716206 | -0.824225292 |
| M00079\|Keratan sulfate degradation | 0.645344898 | 0.754034565 | -0.209825893 | -0.85190578 |
| M00044\|Tyrosine degradation, tyrosine => homogentisate | 0.645004719 | 0.754034565 | -0.206336229 | -0.853680532 |
| M00793\|dTDP-L-rhamnose biosynthesis | 0.643509206 | 0.754034565 | -0.136781369 | -0.865120008 |
| M00223\|Phosphonate transport system | 0.602701629 | 0.735163525 | -0.174724174 | -0.887542125 |
| M00170\|C4-dicarboxylic acid cycle, phosphoenolpyruvate carboxykinase type | 0.587271496 | 0.732439731 | -0.22580172 | -0.899236079 |
| M00122\|Cobalamin biosynthesis, cobinamide => cobalamin | 0.531279557 | 0.677839435 | -0.170744696 | -0.949637796 |
| M00159\|V-type ATPase, prokaryotes | 0.500599441 | 0.646122534 | -0.182758581 | -0.972882882 |
| M00283\|PTS system, ascorbate-specific II component | 0.433065684 | 0.572687648 | -0.222432828 | -1.02277976 |
| M00242\|Zinc transport system | 0.433385247 | 0.572687648 | -0.170878047 | -1.027144543 |
| M00551\|Benzoate degradation, benzoate => catechol / methylbenzoate => methylcatechol | 0.421718948 | 0.572687648 | -0.304609357 | -1.032711255 |
| M00270\|PTS system, trehalose-specific II component | 0.407718348 | 0.572687648 | -0.261467603 | -1.041272413 |
| M00185\|Sulfate transport system | 0.275159744 | 0.408018534 | -0.228639191 | -1.169978175 |
| M00743\|Aminoglycoside resistance, protease HtpX | 0.275688198 | 0.408018534 | -0.208174417 | -1.174859438 |
| M00333\|Type IV secretion system | 0.264896198 | 0.406208076 | -0.213658493 | -1.183440526 |
| M00497\|GlnL-GlnG (nitrogen regulation) two-component regulatory system | 0.253089737 | 0.395675504 | -0.279361441 | -1.192586292 |
| M00459\|VicK-VicR (cell wall metabolism) two-component regulatory system | 0.250894158 | 0.395675504 | -0.251402402 | -1.194535184 |
| M00276\|PTS system, mannose-specific II component | 0.247356445 | 0.395675504 | -0.218425766 | -1.198846512 |
| M00669\|gamma-Hexachlorocyclohexane transport system | 0.20451843 | 0.345548539 | -0.248499701 | -1.252704966 |
| M00417\|Cytochrome o ubiquinol oxidase | 0.205461294 | 0.345548539 | -0.252128557 | -1.258555138 |
| M00701\|Multidrug resistance, efflux pump EmrAB | 0.194278903 | 0.338220207 | -0.248529853 | -1.262451036 |
| M00010\|Citrate cycle, first carbon oxidation, oxaloacetate => 2-oxoglutarate | 0.182851103 | 0.329392048 | -0.254639358 | -1.283655422 |
| M00060\|Lipopolysaccharide biosynthesis, KDO2-lipid A | 0.170963739 | 0.327189225 | -0.223326753 | -1.299923447 |
| M00050\|Guanine ribonucleotide biosynthesis IMP => GDP,GTP | 0.139482983 | 0.286715021 | -0.201687201 | -1.309628231 |
| M00061\|D-Glucuronate degradation | 0.148808732 | 0.294960164 | -0.253930337 | -1.332402953 |
| M00237\|Branched-chain amino acid transport system | 0.13153128 | 0.275471172 | -0.229852724 | -1.356714589 |
| M00208\|Glycine betaine/proline transport system | 0.084389323 | 0.191167651 | -0.261396453 | -1.447857991 |
| M00452\|CusS-CusR (copper tolerance) two-component regulatory system | 0.049102927 | 0.126754068 | -0.372489944 | -1.565066523 |
| M00320\|Lipopolysaccharide export system | 0.046044706 | 0.123572506 | -0.285204966 | -1.565369254 |
| M00255\|Lipoprotein-releasing system | 0.0241773 | 0.070623166 | -0.304090367 | -1.67628758 |
