# Supplementary material for: Antimicrobial Chemicals Associate with Microbial Function and Antibiotic Resistance Indoors
Source: mSystems. 2018 Dec 11;3(6):e00200-18. doi: 10.1128/mSystems.00200-18 (PMC6290264; doi:10.1128/mSystems.00200-18)
Supplement: TABLE S4 [file sys006182300st4.docx]

| **gene** | **isozyme** | **pval** | **qval** | **ES** | **NES** |
| --- | --- | --- | --- | --- | --- |
| K00209: enoyl-[acyl-carrier protein] reductase / trans-2-enoyl-CoA reductase (NAD+) | fabV | 0.782890473 | 0.782890473 | 0.202674572 | 0.647490682 |
| K02371: enoyl-[acyl-carrier protein] reductase II | fabK | 0.512377894 | 0.768566841 | -0.204101835 | -0.9542536 |
| K00208: enoyl-[acyl-carrier protein] reductase I | fabI | 0.098865911 | 0.296597732 | -0.270341436 | -1.429338339 |
